# Randomized clinical trial of bedside ultrasound among patients with abdominal pain in the emergency department: impact on patient satisfaction and health care consumption

**DOI:** 10.1186/1757-7241-17-60

**Published:** 2009-11-27

**Authors:** Anna Lindelius, Staffan Törngren, Laila Nilsson, Hans Pettersson, Johanna Adami

**Affiliations:** 1 Karolinska Institutet, Department of Clinical Science and Education, Södersjukhuset, Stockholm, Sweden; 2 Department of Surgery, Stockholm South General Hospital, Stockholm, Sweden; 3 Karolinska Institutet, Clinical Epidemiology Unit, Stockholm, Sweden

## Abstract

**Background:**

Previous research shows that surgeon-performed ultrasound for patients presenting with abdominal pain in the emergency department leads both to higher diagnostic accuracy and to other benefits. We have evaluated the level of patient satisfaction, health condition and further health care consumption after discharge from the emergency department.

**Methods:**

A total of 800 patients who attended the emergency department for abdominal pain were randomized to surgeon-performed ultrasound or not as a complement to standard examination. All patients were interviewed by telephone six weeks after the visit to the emergency department using a structured questionnaire including information about health condition, satisfaction and medical examinations. A regional health register was used to check health care consumption over two years and mortality was checked for in the personal data register.

**Results:**

We found a higher self-rated patient satisfaction in the ultrasound group when leaving the emergency department. After six weeks the figures were equal. There were fewer patients in the ultrasound group with completed or planned complementary examinations after six weeks (31.1%) compared with the control group (41.4%), p = 0.004. There was no difference found in the two-year health care consumption or mortality between the groups.

**Conclusion:**

For patients with acute abdominal pain, bedside ultrasound examination is related to higher satisfaction and decreased short-term health care consumption. No major effects were revealed when evaluating effects on a long-term basis, including mortality. The previously proven benefit together with the lack of adverse effects from the method makes ultrasound well worth considering for implementation in emergency departments.

**Trial registration:**

The study has been registered in ClinicalTrials.gov ID NCT00550511.

## Background

Ultrasound (US) performed by surgeons or emergency physicians in the emergency department (ED) is increasing worldwide [1-6]. However, the method is debated [2] and in many countries, like Sweden, US is usually performed by a radiologist on request from the physician in the ED.

Studies have been able to show the benefits of a system with surgeon-performed US for patients with abdominal pain in the ED including higher diagnostic accuracy, lower rate of admission, decreased number of further performed examinations and earlier decision regarding surgery [1,7-11]. Long-term effects and patient satisfaction have however not been evaluated to a great extent. We have found one previous study reporting high patient satisfaction when emergency physicians examine patients presenting with abdominal pain with US at the ED. In this study they showed equal satisfaction rates regarding examination by a radiologist or an emergency physician [12].

The aim of this study was to evaluate patient satisfaction and the effects on health condition and health care consumption of the US examination on a short- and a long-term basis. In a previous study we showed that the number of subsequent US examinations were less if the patients had been examined with surgeon-performed US at the ED [10]. In this study we have studied consequences on a short term and long term basis.

We examined the level of patient satisfaction at discharge from the ED and six weeks after the visit. We have also quantified health care consumption and evaluated health condition at six weeks and two years following the ED visit.

## Methods

The methods have been described elsewhere and are therefore summarized briefly [11].

The study was conducted between February 2004 and June 2005 at the ED of Stockholm South General Hospital, a public general hospital with 505 beds and with a catchment area of about 600,000 inhabitants. During this time period a total number of about 11,300 patients attended the ED for abdominal pain.

Nine surgeons, all with at least two years experience of surgery after completed internship, took part in the study. The surgeons attended a one-week course led by a specialist in US examination. This was followed by three weeks training in the radiological department in abdominal US, under the guidance of an ultrasound specialist.

All patients, 18 years or older, admitted to the ED for abdominal pain were considered eligible to participate in the study. The exclusion criteria were: pregnancy, previously diagnosed abdominal condition (a known condition causing the actual pain for which the patient is admitted), acute conditions needing immediate care, inability to communicate with the investigator, severe drug or alcohol addiction and dementia. The study surgeon assessed the patients for participation in the study and included them after informed consent.

A total of 800 patients were enrolled for the study. After inclusion, the patients were examined by the study surgeon. Medical history was taken, and clinical examination and routine laboratory testing were performed. After that a sealed randomization envelope was opened randomizing the patient to US performed by the study surgeon or not. If randomized to the US group, the examination was performed with one out of two handheld, 2,5-5 MHz or 4,3-6 MHz, curved array transducers (B-K medical, Denmark, Hawk 2102, transducers type 8665 and 8802) screening the entire abdomen. The two groups were subsequently managed according to clinical routine as decided by the study surgeon.

Before leaving the ED the patients were asked to anonymously indicate their satisfaction with the visit on a ten-grade visual analogue scale where 0 represents the lowest satisfactory level and 10 the highest level. This paper was sealed by the patient and handed over to the ED staff.

### Short-term follow-up

Four to six weeks after their first visit, all patients received a telephone call from the study nurse. The nurse followed a structured interview questionnaire including questions on health condition, performed and planned examinations after discharge and consultations of other health care providers. The patient was also asked to report his or her self-rated level of satisfaction with the emergency visit on a ten-grade scale where 0 represented the lowest level of satisfaction and 10 the highest. The study nurse was blinded as regards which group the patient belonged to.

### Long-term follow-up

In our regional registry, containing all health contacts in Stockholm with public health care providers, we followed up all patients during a two-year period after the ED visit. On a special case report form, a study nurse recorded all out-patient visits and in-patient admissions during the time period. From the same registry we also recorded radiological examinations and endoscopies within two years of the first visit. We excluded medical care that was obviously not related to the ED visit for abdominal pain, such as hearing and vision examinations, dermatology and medical treatment related to pregnancy and delivery. Mortality was checked for in the personal data register. The study nurse was blinded to which randomization group the patient belonged to.

### Sample size

The sample size was calculated on the basis of the primary outcome of the study, diagnostic accuracy, presented in an earlier article [11]. Thus, the sample size was calculated to detect a nine-percentage points difference for a proportion between the control and the ultrasound groups (specifically 70% versus 79%). It would be necessary to have 400 patients in each group to detect a difference of this size with 80% power at 5% significance level, two-tailed. We used SamplePower 2.0 to perform the sample size calculation

### Ethical considerations

The study was approved by the Institutional Review Board at Karolinska Institutet, Stockholm, Sweden (Dnr 216/03 and 2007/727-32). The study has been registered in ClinicalTrials.gov ID NCT00550511.

### Statistical analysis

The Mann Whitney U test was used to compare the medians between the intervention group and the control group regarding patient satisfaction and health care consumption at the two-year follow-up. In all other comparisons we used the Chi-square test to compare the proportions between the groups. All analyses were performed according to intention-to-treat. The results were regarded as significant if *p *was less than 0.05, two-tailed. SPSS 14.0 was used for statistical analysis.

## Results

### Participation

**Figure 1:**
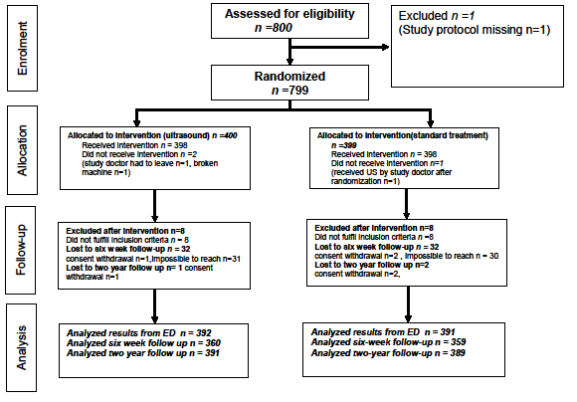
**Study Flow Chart**.

A study flow chart is shown in Figure 1. A total number of 392 patients in the US group and 391 patients in the control group were available for analysis from the ED, including patient satisfaction measure and baseline characteristics. 360 patients in the US group and 359 patients in the control group were available for the six-week follow-up analysis. For the two-year follow-up, 391 patients in the US group and 389 patients in the control group were included in the analysis.

### Characteristics at baseline

The groups were similar concerning all background factors, except for referral pattern. More patients were referred to the ED in the group not undergoing US (Table 1).

**Table 1:** Baseline characteristics of patients with abdominal pain at the Emergency Department enrolled in this study

Characteristics	Ultrasound (n = 392)		Not ultrasound (n = 391)	
	
	mean (SD)	n (%)	mean (SD)	n (%)
Age	47 (20)		48 (19)	

Gender				
*Male*		160 (40.8)		171 (43.7)

*Female*		232 (59.2)		220 (56.3)

Height	172 (9)		172 (10)	

Weight	73 (16)		73 (16)	

BMI (Body Mass Index)	24,8 (4.5)		24.8 (4.3)	

Abdominal-related comorbidity		76 (19.4)		78 (19.9)

Comorbidity related to heart or diabetes		66 (16.8)		74 (18.9)

History of abdominal malignancy		6 (1.5)		12 (3.1)

History of other malignancy		11 (2.8)		14 (3.6)

Other comorbidity		132 (33.7)		123 (31.5)

Admission for abdominal pain within one year		124 (32.0)		137 (35.3)

Referral for admission		92 (24.4)		126 (32.9)

Duration of pain				
*0-8 hours*		44 (14.8)		43 (14.4)

*8-24 hours*		99 (33.2)		97 (32.4)

*>24 hours*		147 (49.3)		151 (50.5)

*Cannot answer*		8 (2.7)		8 (2.7)

Actual VAS (of pain)	4.3 (2.8)		4,4 (2.6)	

Maximal recall VAS (of pain)	7.6 (2.6)		7,6 (1.8)	

Temperature	37.0 (0.8)		37.0(0.7)	

Affected general condition		90 (23.3)		74 (19.1)

Tenderness		338 (86.4)		347 (89.2)

Rigidity		51 (13.1)		49 (12.6)

Palpable mass		23 (5.9)		29 (7.5)

VAS (of pain) = Visual Analogue Scale (scale 0-10. 0 represents no pain at all, 10 represents unbearable pain)

### Patient satisfaction

The self-rated patient satisfaction when leaving the ED was slightly, but significantly, higher in the US group. At the six-week follow-up the patient satisfaction measured was equal in both groups (Table 2).

**Table 2:** Patient satisfaction (VAS)

	Ultrasound *			Not ultrasound **			
	Mean	Median	SD	Mean	Median	SD	p-value
At Emergency Department	8.9	9.5	1.4	8.7	9.2	1.6	0.005
At six-week follow-up	8.1	8.0	1.9	8.0	8.0	2.1	0.958

VAS (of satisfaction) = Visual Analogue Scale (scale 0-10. 0 represents the lowest satisfaction level, 10 represents highest satisfaction level). Min value 0 and max value 10 at ED and at six-week follow-up in both groups.

*n = 373 at ED, n = 356 at follow-up (missing data in 19 patients at ED and 4 at follow-up)

**n = 364 at ED, n = 353 at follow-up (missing data in 27 patients at ED and 6 at follow-up)

### Short-term follow-up

**Figure 2:**
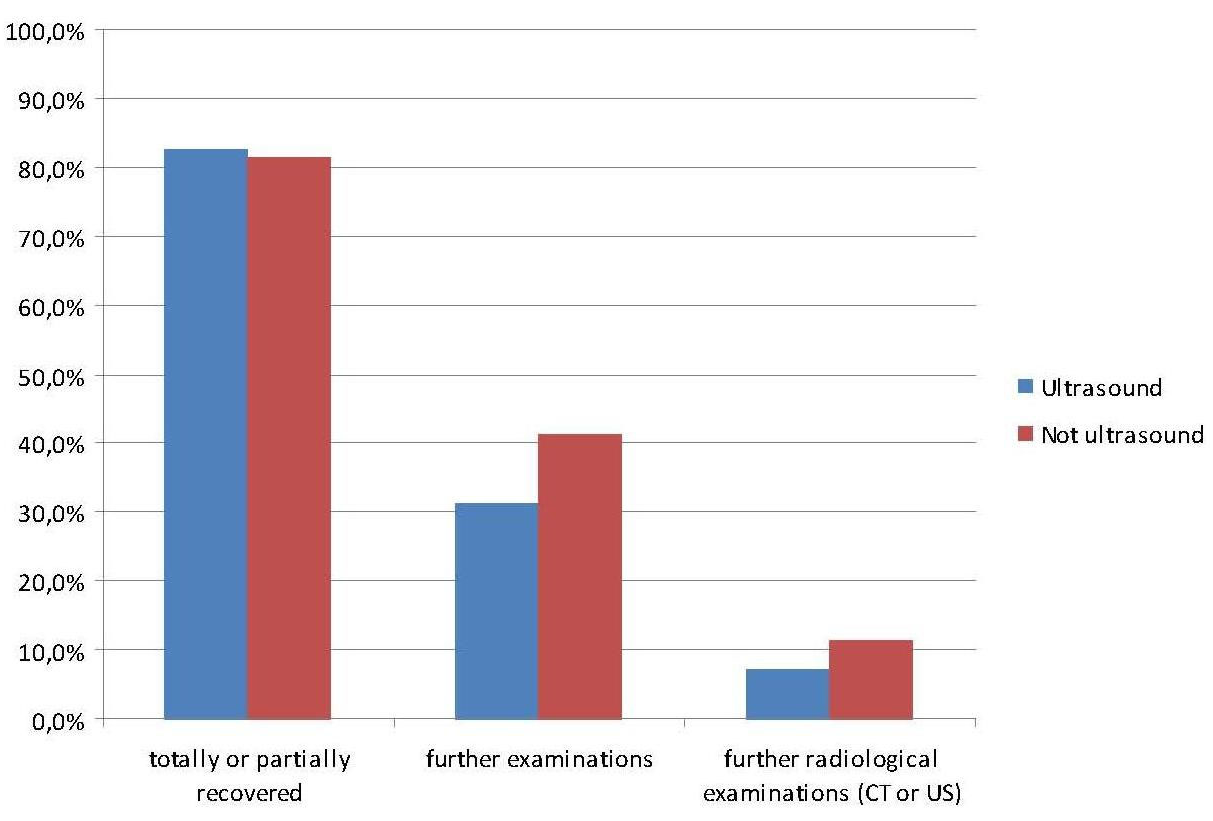
**Rate of recovery and subsequent examinations at six week follow up comparing patients that underwent US with those who did not undergo US at the ED**.

31.1% of the patients in the US group had completed or planned complementary examinations after the ED visit compared to 41.4% in the control group (p = 0.004). When analyzing examinations separately there was only a significant difference in US examinations and colonoscopies with a higher frequency of these examinations in the control group. Self-reported health condition was equal in both groups (Table 3). These results are also illustrated in Figure 2.

**Table 3:** Health condition and health care consumption at six-week follow-up

	Ultrasound n = 360 n(%)	Not ultrasound n = 359 n(%)	p-value
Further examinations (performed or planned)*	111(31.1)	146(41.4)	0.004

Planned	40(11.2)	60(17.0)	

Completed	71(19.9)	86(24.4)	

Not planned	246(68.9)	206(58.6)	

Computer tomography	12(3.4)	12(3.4)	0.989

Ultrasound	13(3.7)	29(8.2)	0.010

Laboratory tests	3(0.8)	1(0.3)	0.319

Gastroscopy	16(4.5)	15(4.2)	0.867

Colonoscopy	20(5.6)	38(10.7)	0.013

Urography	19(5.3)	23(6.5)	0.512
Other examinations	46(12.9)	58(16.4)	0.192

Doctor consultation	88(24.7)	77(21.6)	0.329

Health condition**			0.984
totally well	218(60.7)	215 (59.9)	

partly well	78(21.7)	77(21.4)	

not well can not tell	59(16.4) 4(1.1)	63(17.5) 4(1.1)	
can not tell	4(1.1)	4(1.1)	

*Missing data in 3 patients in US group and 7 patients in not US group

** Missing data in 1 patient in US group

### Long-term follow-up

There was no significant difference between the groups concerning health care consumption during two years after the ED visit (Table 4).

**Table 4:** Health care consumption at two-year follow-up

	Ultrasound (n = 388*)			Not ultrasound (n = 383**)			
	
	Median (min-max)	Mean	SD	Median (min-max)	Mean	SD	p-value
Number of out-patient admissions	5.0 (0-500)	13.8	31.6	7.0 (0-183)	13.5	19.9	0.220

Number of out-patient radiological examinations	1.0 (0-11)	1.4	2.0	1.0 (0-16)	1.5	2.1	0.294

Number of out-patient endoscopies	0.0 (0-3)	0.2	0.5	0.0 (0-3)	0.2	0.5	0.108

Number of in-patient admissions	0.0 (0-18)	1.1	2.3	0.0 (0-14)	1.1	2.2	0.774

Total amount of hospital days	0.0 (0-462)	6.0	26.3	0.0(0-470)	8.7	35.6	0.733

*Missing data for 3 patients

**Missing data for 6 patients

### Mortality

There was no significant difference between the groups regarding mortality (Table 5). The three deaths in the US group at six-week follow-up were not associated with US: an 80-year-old woman that was admitted with acute leukemia, transferred to another hospital and died there two days later; a 68-year-old woman who died of metastatic lung carcinoma three weeks later; and a 93-year-old woman who died of acute myocardial infarction at a geriatric clinic five days after the ED visit.

**Table 5:** Mortality

	Ultrasound n = 391 n (%)	Not ultrasound n = 389 n (%)	p-value
Six-week follow-up	3 (0.8)	0 (0.0)	0.083
Two-year follow-up	17 (4.3)	22 (5.6)	0.407

## Discussion

This study is the first randomized study assessing patient satisfaction and the long-term effects as regards health care consumption when using US for diagnosis of abdominal pain at the ED. We found a small, but still significant, increase in patient satisfaction directly after the ED visit. Factors shown to be related to patient satisfaction at the ED include actual and perceived waiting time, numbers of treatments in the ED, provider-patient interactions and the adequacy of information provided, age, triage status and explanation of causes of problem and tests [13-15]. A possible explanation for our results with higher satisfaction for the US group at the ED could though be the additional examination performed and possibly subsequently a better patient-provider interaction with a better explanation of the patient's problem with the help of the US examination results. More subsequent examinations were performed in the group not receiving US at the ED in this study, which may have had an impact on patient satisfaction. If patient satisfaction had been measured immediately after the US performed by the surgeon, before decision had been taken about complementary examinations, it might have been even higher. Our aim was to measure the patient satisfaction concerning all aspects of the introduced method of bedside US and therefore we estimated the overall satisfaction rate which we believe reflects this. Time consumption for the groups were reported in an earlier paper [10]. Since the length of stay at the ED was about the same in both groups (about 4.5 hours) the waiting time at the ED would probably not affect the rates of patient satisfaction. Background factors as age and BMI were equal for both groups and do not interfere with the results. Though patient satisfaction was slightly higher at ED when US was used, the rates did not differ at six-week follow-up but were still quite high, in line with another study examining satisfaction rates after US examinations for abdominal pain [12].

Mortality rates for patients visiting the ED are shown to be fairly high, especially for frequent ED users [16-18]. Health care consumption is also shown to be high in this group [16]. One previous randomized study that examined the six-month mortality rate did not show any difference between patients with abdominal pain examined with early CT or not [19]. In our study we did not find any difference in mortality either. Two-year health care consumption was also equal between the groups. On a short-term basis there were however fewer requested complementary examinations in the group where US was performed.

Previous studies have shown that bedside surgeon-performed US can increase diagnostic accuracy of abdominal pain [1,11]. Moreover, other benefits of bedside US have been reported, such as decreased admission frequency, less need of complementary examinations and shorter time for surgery with the use of surgeon-performed US at the bedside when a patient presents with abdominal pain [7-10].

The results shown in this study seem to support routine use of the method in the ED. Bedside US is an easy examination without any known side-effects [20,21] With proved benefits, higher patient satisfaction and no negative long-term effects, we believe that US is safe to recommend. Taking into account that abdominal pain is a common reason for seeking medical care all over the world [22-24], this easy examination would save money and hospital beds and give radiologists more time to perform other examinations. There are also benefits for the patient who does not have to come back for further examinations to the same extent after leaving the ED.

The strengths of this study are the randomization procedure and the large number of comparable patients included. We also have an almost complete follow-up of the patients.

One weakness is the imprecision of the information in the regional health care registry. The medical care providers are supposed to give complete information to the registry but we have noted some inaccuracy. For example more than one registration was found for the same day. We were unable to validate the data afterwards to be sure that only conditions related to the actual ED visit were recorded. However, since the study is randomized, any misclassification would not lead to any bias in the comparisons between the groups and therefore not affect our conclusion. We have though no reason to doubt that the data on hospital care and the short-term follow-up by the blinded nurse are correct.

## Conclusion

This study shows no long-term side-effects on health care consumption and no increased mortality related to examination with surgeon-performed US in patients presenting in the ED with abdominal pain. The immediate patient satisfaction is slightly higher in the US group and health care consumption lower in the short term. Therefore, taking into consideration other benefits, we believe that implementation of bedside US in the ED improves management of the patients.

## Competing interests

The authors declare that they have no competing interests.

## Authors' contributions

AL conceived and designed the study together with ST and JA. LN collected the six week and two year follow up data. AL and HP performed the data analysis. AL drafted the manuscript. All authors interpreted data and critically revised the manuscript.
